# Defective erythropoiesis in primary myelofibrosis associated with a chromosome 11 abnormality.

**DOI:** 10.1038/bjc.1991.255

**Published:** 1991-07

**Authors:** W. N. Patton, C. M. Bunce, S. Larkins, G. Brown

**Affiliations:** Department of Haematology, Medical School, University of Birmingham, UK.

## Abstract

**Images:**


Br. J. Cancer (1991), 64, 128-131 © Macmillan Press Ltd., 1991

### Defective erythropoiesis in primary myelofibrosis associated with a chromosome 11 abnormality

W.N. Patton[1], C.M. Bunce[2], S. Larkins[3] & G. Brown[2]

Departments of [1]Haematology and [2]Immunology, The Medical School, University of Birmingham, Birmingham B15 2TT and [3]Department of Cytogenetics, Birmingham Maternity Hospital, Birmingham B15 2TH, UK.

**Summary** A case of primary myelofibrosis was identified with a previously unreported complex karyotype with two abnormal clones in addition to a proportion of normal cells: 46,XY,−2,−11,+der(2)t(2;11)(q24/31;q13),+mar and 45,XY,−2,−11,+der(2)t(2;11)(q24/31;q13),+mar,−17,del(7q). Study of circulating committed progenitors from this patient consistently showed (1) an absence of erythroid progenitors which is uncommon and (2) greatly increased granulocyte-monocyte progenitors (CFU-GM) which is generally observed in myelofibrosis. Further study showed that peripheral blood mononuclear cells co-cultured with irradiated normal bone marrow stroma generated increased numbers of CFU-GM compared with controls but failed to generate erythroid progenitors, providing evidence for an intrinsic defect in erythropoiesis. Only once previously has the absence of erythroid progenitors in primary myelofibrosis been studied in relation to cytogenetic abnormalities. This case also revealed a complex karyotype which, however, shared with our case a defect on chromosome 11. The identification of two cases of primary myelofibrosis which lack committed erythroid progenitor cells and which show in common a chromosomal defect on chromosome 11 point to the existence of genes on this chromosome which play a key role during erythropoiesis.

Primary myelofibrosis (PMF) is a form of chronic myeloproliferative disease characterised by bone marrow fibrosis, hepatosplenomegaly and the presence of leucoerythroblastic changes and tear drop poikilocytes in the peripheral blood. The finding of G-6-PD isoenzyme restriction in patients' peripheral blood cells, but not fibroblasts and the demonstration of clonal cytogenetic abnormalities in pluripotent haemopoietic progenitor cells support the hypothesis that the primary defect is a clonal expansion of pluripotent haemopoietic progenitor cells associated with reactive marrow fibrosis (Jacobson *et al.*, 1978; Ruutu *et al.*, 1983; Sato *et al.*, 1986; Sugiyama *et al.*, 1989). Reported cytogenetic aberrations have been varied but abnormalities of chromosome 13 have been the most common (Borgstrom *et al.*, 1984; Johnson *et al.*, 1985). PMF is also associated with extramedullary haemopoiesis and increased levels of circulating haemopoietic progenitors including multilineage progenitors (CFU-GEMM), megakaryocyte progenitors (CFU-Mk), erythroid progenitors (BFU-E) and granulocyte-macrophage progenitors (CFU-GM). Many cases have even shown erythropoietin independent erythroid colony formation (Hibbin *et al.*, 1984; Carlo-Stella *et al.*, 1987).

We report on a case of PMF in whom defective erythropoiesis, characterised by the absence of circulating erythroid progenitors and the inability to generate erythroid colonies on irradiated normal marrow stroma, was associated with a complex cytogenetic abnormality. Comparison of the karyotype in this case with that observed in a previously reported case in whom erythropoiesis was defective (Partenen *et al.*, 1982) reveals a common abnormality involving chromosome 11. The significance and possible linkage between defective erythropoiesis and the karyotypic abnormality is discussed.

### Case report

A 71 year old man presented with anaemic symptoms, 8 cm hepatomegaly and 12 cm splenomegaly. His Hb was $43\ g\ l^{-1}$, Wbc $2.8 \times 10^9\ l^{-1}$ with neutrophils $2.2 \times 10^9\ l^{-1}$ including hypersegmented forms, lymphocytes $0.3 \times 10^9\ l^{-1}$, monocytes $0.2 \times 10^9\ l^{-1}$, myelocytes $< 0.1 \times 10^9\ l^{-1}$, blasts $0.1 \times 10^9\ l^{-1}$, normoblasts 1 per 100 w.b.c. and platelet count $95 \times 10^9\ l^{-1}$. His blood film showed tear drop poikilocytes but infrequent normoblasts which were rarely seen on subsequent blood films. His reticulocyte count was $33 \times 10^9\ l^{-1}$ and both the serum and red cell folate were reduced. Bone marrow aspiration was unsuccessful and a trephine biopsy was hypercellular showing marked granulocytic hyperplasia, absent normoblasts, megakaryocytes of increased size with irregular nuclei and increased reticulin fibrosis, consistent with features in cases reported by Barosi *et al.*, 1983. He was initially supported with folic acid, allopurinol and blood transfusion and was subsequently treated with hydroxyurea and oxymethalone. He remained transfusion dependent but after 7 months developed an increasing leukocyte count with an increasing proportion of blast cells which was refractory to therapy with hydroxyurea. He died 9 months after presentation having followed a course similar to patients reported by Bentley *et al.*, 1977.

Correspondence: W.N. Patton, Department of Haematology, The Medical School, University of Birmingham, Birmingham B15 2TT.
Received 16 November 1990; and in revised form 21 January 1991.

### Materials and methods

### Cells

Peripheral blood mononuclear cells (PBMNC) from the above case collected into preservative free heparin were separated on ficoll-hypaque (Pharmacia, UK) and the interface cells were washed three times in Iscove's modified Dulbecco's medium (IMDM) (Gibco, UK).

### *Assays for CFU-GM and BFU-E*

Fresh PBMNC were assayed for their ability to form colonies in semi-solid medium using a method similar to that described by Ash *et al.*, 1981. For a CFU-GM assay, $2-8 \times 10^4$ PMF PBMNC were seeded in duplicate in a 1.0 ml volume of IMDM containing 1.2% methylcellulose (Sigma), 20% foetal calf serum (FCS, Gibco), 1% deionised bovine serum albumin (Sigma), $5 \times 10^{-5}$ M 2-mercaptoethanol (Sigma), 5% phytohaemagglutinin stimulated leucocyte conditioned medium (PHA-LCM) and $100\ U\ ml^{-1}$ penicillin − $50\ \mu g\ ml^{-1}$ streptomycin (Gibco). Colonies containing >40 cells were enumerated at day 12. For assessment of BFU-E an identical system containing erythropoietin (Eprex, Cilag UK) at

$3\,U\,ml^{-1}$ was used, the cells were plated at densities of $0.5-1.0 \times 10^5\,ml^{-1}$ and the plates read after 14 days. Fresh fractionated human foetal liver cells (Toksoz & Brown, 1984) and normal bone marrow cells were used as positive controls. The assays were performed on three different occasions, firstly at diagnosis and on two other occasions when hydroxyurea therapy had been stopped 96 h previously.

*Initiation of haemopoiesis on irradiated stroma from long term bone marrow cultures*

Fresh PBMNC were also tested for their ability to initiate haematopoiesis on irradiated stroma from long term bone marrow cultures (LTC). A 10 ml long term bone marrow culture containing $2 \times 10^7$ marrow buffy coat cells from a normal marrow donor was established and maintained in medium as previously described (Gartner & Kaplan, 1980). After 30 days nonadherent cells were removed, the adherent cells trypsinised, irradiated with 15 Gray (Co 60 gamma rays, mean energy 1.25 MeV, dose rate $5.3\,Gy\,min^{-1}$) and 3 ml of cells at $1 \times 10^5\,ml^{-1}$ reseeded (i.e. at $3 \times 10^4\,cm^{-2}$) in each of three 35 mm diameter wells of a 6 well tissue culture plate (Nunc). After 5 days incubation, during which a healthy stroma had re-established, 1.5 ml of supernatant was removed from each of the three wells and to each of two wells was added 1.5 ml of LTC medium containing $5.4 \times 10^6$ fresh PMF PBMNC. To the remaining well was added 1.5 ml LTC medium alone to act as a negative control. To each of two other empty 35 mm diameter wells was added $5.4 \times 10^6$ fresh PMF PBMNC in 3 ml of LTC medium to act as a PMF control. The five cultures were fed weekly by removing half of the supernatant and replacing this with fresh LTC medium. After 5 weeks all nonadherent cells were removed and the cells in the adherent layer harvested by trypsinisation. Nonadherent and adherent cells were pooled, washed and assayed for progenitor cells as described above. The total number of cells harvested from each culture was as follows: PMF PBMNC alone $11 \times 10^5$ and $14 \times 10^5$; co-cultures of PBMNC and irradiated stroma $15 \times 10^5$ and $17 \times 10^5$; and irradiated stroma alone $1.4 \times 10^5$ cells. The cells in the stroma alone culture were plated in duplicate at a density of $0.7 \times 10^5\,ml^{-1}$ in an erythroid colony assay. Otherwise cells from the other cultures were plated in triplicate in separate assays for CFU-GM and BFU-E at densities ranging from $1.5 \times 10^5-2.3 \times 10^5\,ml^{-1}$.

*Cytogenetic analysis*

For analysis of peripheral blood cells, trypsin-Giemsa banded slides were prepared from unstimulated cultures which after 24 h were treated with $0.02\,\mu g\,ml^{-1}$ colcemid for either 1 h or 24 h. Metaphases were analysed using standard chromosome criteria. To demonstrate any cytogenetic abnormality in CFU-GM isolated from our patient, pooled GM colonies grown from PBMNC were harvested into IMDM containing 20% FCS and 10% phytohaemagglutinin-leucocyte conditioned medium, and incubated overnight at 37°C in 5% $CO_2$. Colcemid was added and after 1 h and 24 h cells were harvested and processed as described above.

### Results

*Cytogenetic analysis*

Analysis of 20 metaphases from unstimulated cultures of PBMNC showed three cell lines: one metaphase showed a normal male karyotype; 13 cells showed 46 chromosomes with the loss of one chromosome 2 and one 11, the presence of an abnormal chromosome 2 derived from a 2;11 translocation and an additional unidentified marker chromosome (46,XY,−2,−11,+der(2)t(2;11)(q24/31;q13),+mar); and six cells showed the above karyotype along with the loss of one chromosome 17 and a deletion of part of the long arm of one chromosome 7 (45,XY,−2,−11,+der(2)t(2;11)(q24/31;q13),+mar,−17,del(7q)). Analysis of pooled GM colonies grown from PBMNC showed the third karyotype described above in 8/10 metaphases. The other two metaphases described the same karyotype with the addition of another unidentified marker chromosome, implying some further clonal evolution (Figure 1).

*Colony forming ability of PBMNC*

On three separate occasions culture of PBMNC revealed greatly increased numbers of circulating CFU-GM with the complete absence of erythroid colonies (Table I). The nature of GM colonies was confirmed following staining of cytocentrifuged preparations of single colony contents. Positive control erythroid colonies were grown from fractionated human foetal liver cells and from bone marrow cells.

*Initiation of haemopoiesis on irradiated stroma from LTC*

No GM or erythroid colonies were grown from the culture containing irradiated allogeneic normal bone marrow stroma alone and only GM colonies were grown in the other cultures containing PMF PBMNC. Co-cultures containing both irradiated stroma and PMF PBMNC yielded much greater numbers of GM colonies than cultures containing PMF PBMNC alone whether numbers were expressed in terms of total CFU per culture or per $10^5$ inoculating cells (Table II). Rowanowsky stained cytospins of cells taken from cultures at the time of clonogenic assay did not show any cells of the erythroid lineage.

### Discussion

The main observations from this case study are the demonstration of absent circulating erythroid progenitors in PMF, an uncommon event, and its association with a previously unreported karyotypic abnormality.

Circulating haemopoietic progenitors, particularly CFU-GM, are usually greatly increased in PMF. Studies of circulating erythroid progenitors have shown normal or increased levels in 18/18 cases studied by Carlo-Stella *et al.*, 1987, 17/18 cases studied by Partenen *et al.*, 1982 and 2/2 cases studied by Douer *et al.*, 1983. However, cases of PMF have been reported in which erythroid progenitor cells are absent. In the study of Hibbin and co-workers erythroid progenitors were absent in four splenectomised patients (Hibbin *et al.*, 1984) and Croizat *et al.*, 1983 observed an absence of erythroid progenitors in four patients, two of which had been splenectomised. The absence of circulating erythroid progenitors in PMF patients is not invariably linked to splenectomy since the patient reported in this study and the one identified by Partenen and co-workers with absent BFU-E (Partenen *et al.*, 1982) were not splenectomised. Furthermore, the two larger studies of 18 patients showed normal or increased levels of circulating BFU-E in many splenectomised patients (Partenen *et al.*, 1982; Carlo-Stella *et al.*, 1987).

Many observations support the fact that circulating committed progenitors in PMF largely arise from and circulate from the spleen (Douay *et al.*, 1987). Thus, in the patient reported in this study the failure to detect circulating erythroid progenitors is due to either a defect in the capacity of haemopoietic stem cells to undergo commitment to the erythroid lineage or the ability of committed erythroid progenitors to undergo erythropoiesis or that erythroid progenitors are generated and sequestered in the spleen.

An intrinsic defect in erythropoiesis is the most likely explanation for the following reasons. Douay and co-workers have shown that, in PMF patients, CFU-GM can be maintained in liquid suspension culture in the absence of a substantial stromal layer over a 10-week period and concluded that primitive stem cells circulated in PMF patients (Douay *et al.*, 1987). In our study, PBMNC were co-cultured with normal marrow stroma to maintain circulating primitive stem cells and assess whether they were able to give rise to ery-

**Figure 1** G-banded karyotype from the GM colony culture: 46,XY,−2,−11,+der(2)t(2;11)(q24/31;q13),+mar1, −17,del(7q),+mar2.

**Table I** Number of colony forming cells grown from PMF PBMNC and normal cells

| | *PMF PBMNC* | | *Foetal Liver* | | *Bone marrow* | |
|---|---|---|---|---|---|---|
| | *CFU ml$^{-1}$ of blood* | | *CFU per $10^5$ cells* | | | |
| *Time point* | *CFU-GM* | *BFU-E/CFU-E* | *CFU-GM* | *BFU-E* | *CFU-GM* | *BFU-E* |
| 1 | 2,644 | 0 | 206 | 153 | 33 | 22 |
| 2 | 7,912 | 0 | 244 | 166 | 70 | 28 |
| 3 | 7,605 | 0 | 300 | 90 | 27 | ND |

ND = not done.

**Table II** Mean number (± s.e.m.) of CFU isolated after culture of PMF PBMNC on irradiated bone marrow stroma

| | *CFU-GM* | | |
|---|---|---|---|
| *Culture condition* | *Per $10^5$ cells* | *Per total culture* | *BFU-E/CFU-E* |
| Stroma alone | 0 | 0 | 0 |
| PMF cells alone | 14 (1.1), 11 (1.7) | 156 (12), 101 (16) | 0 |
| PMF cells + stroma | 27 (2.1), 20 (0.7) | 405 (31), 348 (11) | 0 |

throid progenitors in a normal and appropriate microenvironment. Erythroid progenitor cells were not generated and the existence of circulating stem cells was supported by the generation of increased numbers of CFU-GM following incubation of PBMNC with marrow stroma as compared with control PMF PBMNC cultured in the absence of stroma. The failure to detect circulating erythroid progenitors for technical reasons can be excluded since the assays were controlled by the demonstration of erythroid progenitors in appropriate numbers from foetal liver and from bone marrow cells.

Of those PMF patients previously studied with absent circulating erythroid progenitors, cytogenetic data is available for only one case which exhibited a complex karyotype – 47,XX,5q−.11q−,−20,+mar 1,+mar 2 (Partenen *et al.*, 1982). However, a common abnormality involving the long arm of chromosome 11 between this and our case is of interest and suggests a causal relationship between an abnormality in the long arm of chromosome 11 and defective erythropoiesis. An abnormality at 11q13 has been reported once previously in a case of PMF, though whether erythropoiesis was defective in this case in unknown (Sessarego *et al.*, 1983). A review of the literature with regard to oncogene associations at the relevant breakpoints in the case reported in this study reveals that the proto-oncogene SEA (S13 avian erythroblastosis oncogene homolog) maps to the 11q13 region (Williams *et al.*, 1987; Nordenskjold *et al.*, 1989; Hayman *et al.*, 1985). The involvement of this oncogene should be explored at the molecular level.

In conclusion, the association between a distinct cytogenetic abnormality on chromosome 11 and the absence of committed erythryoid progenitor cells in PMF patients is worthy of further study. Cytogenetic analysis of additional patients with an absence of circulating erythroid progenitors might reveal a common chromosome abnormality and point to the location of genes which encode key intrinsic regulators of erythoid lineage development. Furthermore, studies of lesions in the generation of various committed progenitor cells in PMF patients in relation to possible karyotypic abnormalities offers a useful approach to the analysis of the chromosomal location and organisation of genes which control the generation and differentiation of haemopoietic progenitor cells.

We are grateful to Mr P. Anderson of the Department of Immunology, University of Birmingham for assistance in the irradiation of stromal cells. We also thank the Leukaemia Research Fund for support of research in our laboratory. W.N.P. is a clinical research fellow supported by the United Birmingham Hospitals Trust Fund.

### References

ASH, R.C., DETRICH, R.A. & ZANJANI, E.D. (1981). Studies of human pluripotential haemopoietic stem cells (CFU-GEMM) *in vitro*. *Blood*, **58**, 309.

BAROSI, G., BARALDI. A., CAZZOLA, M., SPRIANO, P. & MAGRINI, U. (1983). Red cell aplasia in myelofibrosis with myeloid metaplasia. A distinct functional and clinical entity. *Cancer*, **52**, 1290.

BENTLEY, S.A., MURRAY, K.H., LEWIS, S.M. & ROBERTS, P.D. (1977). Erythroid hypoplasia in myelofibrosis: a feature associated with blastic transformation. *Br. J. Haematol.*, **36**, 41.

BORGSTROM, G.H., KNUUTILA, S., RUUTU, T., PAKKALA, A., LAHTINEN, R. & DE LA CHAPELLE, A. (1984). Abnormalities of chromosome 13 in myelofibrosis. *Scand. J. Haematol.*, **33**, 15.

CARLO-STELLA, C., CAZZOLA, M., GASNER, A. & 6 others (1987). Effects of recombinant alpha and gamma interferons on the *in vitro* growth of circulating haematopoietic progenitor cells (CFU-GEMM, CFU-Mk, BFU-E and CFU-GM) from patients with myelofibrosis and myeloid metaplasia. *Blood*, **70**, 1014.

CROIZAT, H., AMATO, D., MCLEOD, D.L., ESKINAZI, D. & AXELRAD, A.A. (1983). Differences among myeloproliferative disorders in the behaviour of their restricted progenitor cells in culture. *Blood*, **62**, 578.

DOUAY, L., LAPORTE, J.P., LEFRANCOIS, G. & 5 others (1987). Blood and spleen haematopoiesis in patients with myelofibrosis. *Leuk. Res.*, **11**, 725.

DOUER, D., FABIAN, I. & CLINE, M.J. (1983). Circulating pluripotent haemopoietic cells in patients with myeloproliferative disorders. *Br. J. Haematol.*, **54**, 373.

GARTNER, S. & KAPLAN, H.S. (1980). Long term culture of human bone marrow cells. *Proc. Natl Acad. Sci. USA*, **77**, 4756.

HAYMAN, M.J., KITCHENER, G., VOGT, P.K. & BEUG, H. (1985). The putative transforming protein of S13 avian erythroblastosis virus is a transmembrane glycoprotein with an associated protein kinase activity. *Proc. Natl Acad. Sci. USA*, **82**, 8237.

HIBBIN, J.A., NJOKU, O.S., MATUTES, E., LEWIS, S.M. & GOLDMAN, J.M. (1984). Myeloid progenitor cells in the circulation of patients with myelofibrosis and other myeloproliferative disorders. *Br. J. Haematol.*, **57**, 495.

JACOBSON, R.J., SALO, A. & FIALKOW, P.J. (1978). Agnogenic myeloid metaplasia: a clonal proliferation of haematopoietic stem cells with secondary myelofibrosis. *Blood*, **51**, 189.

JOHNSON, D.D., DEWALD, G.W., PIERRE, R.V., LETENDRE, L. & SILVERSTEIN, M.N. (1985). Deletions of chromosome 13 in malignant hematologic disorders. *Cancer Genet. Cytogenet.*, **18**, 235.

NORDENSKJOLD, M., JONES, C.A., GLASER, T., WERELIUS, B., LARSSON, C. & JANSSON, M.C. (1989. Mapping of chromosome 11q probes on somatic cell hybrids. *Cytogenet. Cell Genet.*, **51**, 1054 (abstract).

PARTENEN, S., RUUTU, T. & VUOPIO, P. (1982). Circulating haematopoietic progenitors in myelofibrosis. *Scand. J. Haematol.*, **29**, 325.

RUUTU, T., PARTENEN, S. & KNUUTILA, S. (1983). Clonal karyotype abnormalities in erythroid and granulocyte-monocyte precursors in polycythaemia vera and myelofibrosis. *Scand. J. Haematol.*, **31**, 253.

SATO, Y., SUDA, T., SUDA, J. & 4 others (1986). Multilineage expression of haemopoietic precursors with an abnormal clone in idiopathic myelofibrosis. *Br. J. Haematol.*, **64**, 657.

SESSAREGO, M., AJMAR, F., RAVAZZOLO, R., BIANCHI SCARRA, G.L., GARRE, C. & BOCCACCIO, P. (1983). Coincidence between fragile site expression and interstitial deletion of chromosome 11 in a case of myelofibrosis. *Hum. Genet.*, **63**, 299.

SUGIYAMA, H., ICHIBA, S., OKUNO, Y. & 7 others (1989). Cytogenetic evidence for a clonal disorder involving CFU-GEMM, BFU-E and CFU-C in patients with myeloproliferative disorders. *Nippon Ketsueki Gakkai Zasshi*, **52**, 1022.

TOKSOZ, D. & BROWN, G. (1984). Maintenance of granulocyte-monocyte progenitors in liquid cultures of human fetal liver. *J. Cell. Physiol.*, **119**, 227.

WILLIAMS, B.P., GOODFELLOW, P.N., SHIPLEY, J.M., SPURR, N.K., SMITH, D.R. & HAYMAN, M.J. (1987). Assignment of SEA, a putative human oncogene, to chromosome 11q13. *Cytogenet. Cell Genet.*, **46**, 717 (abstract).


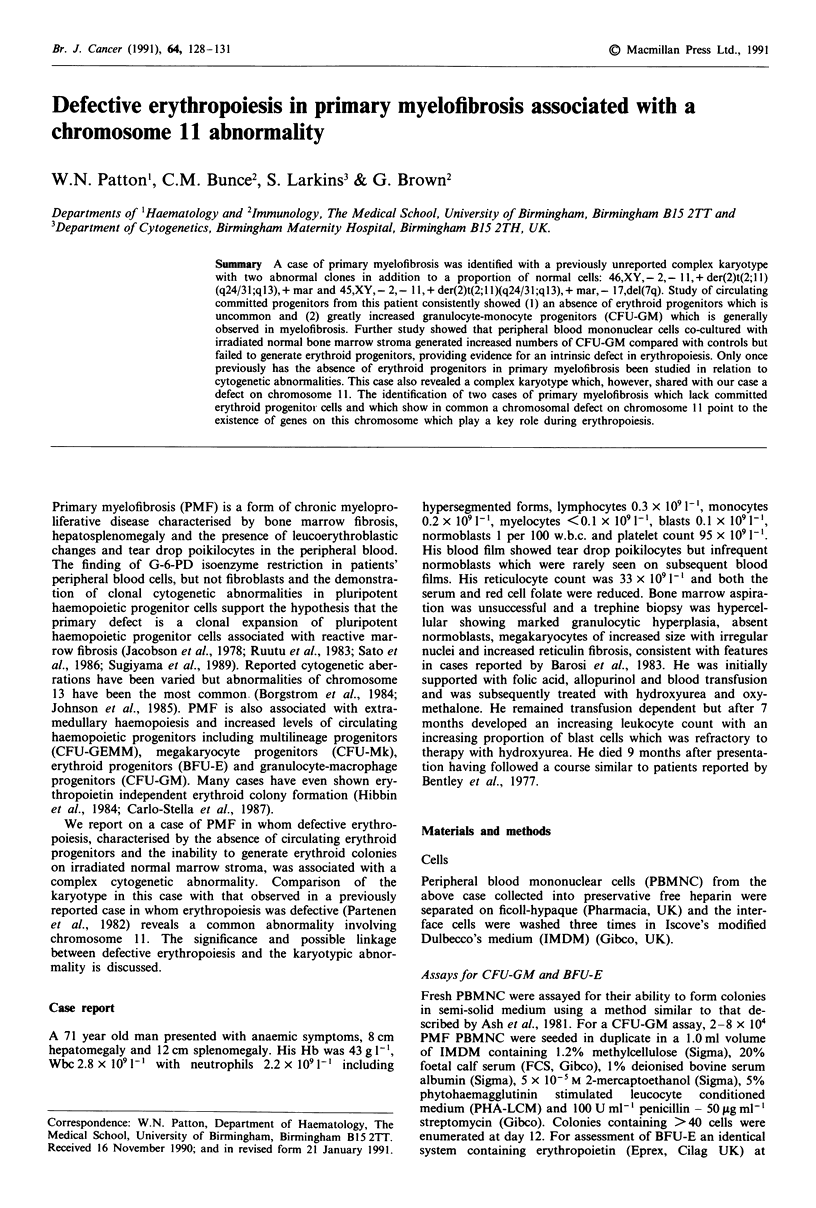

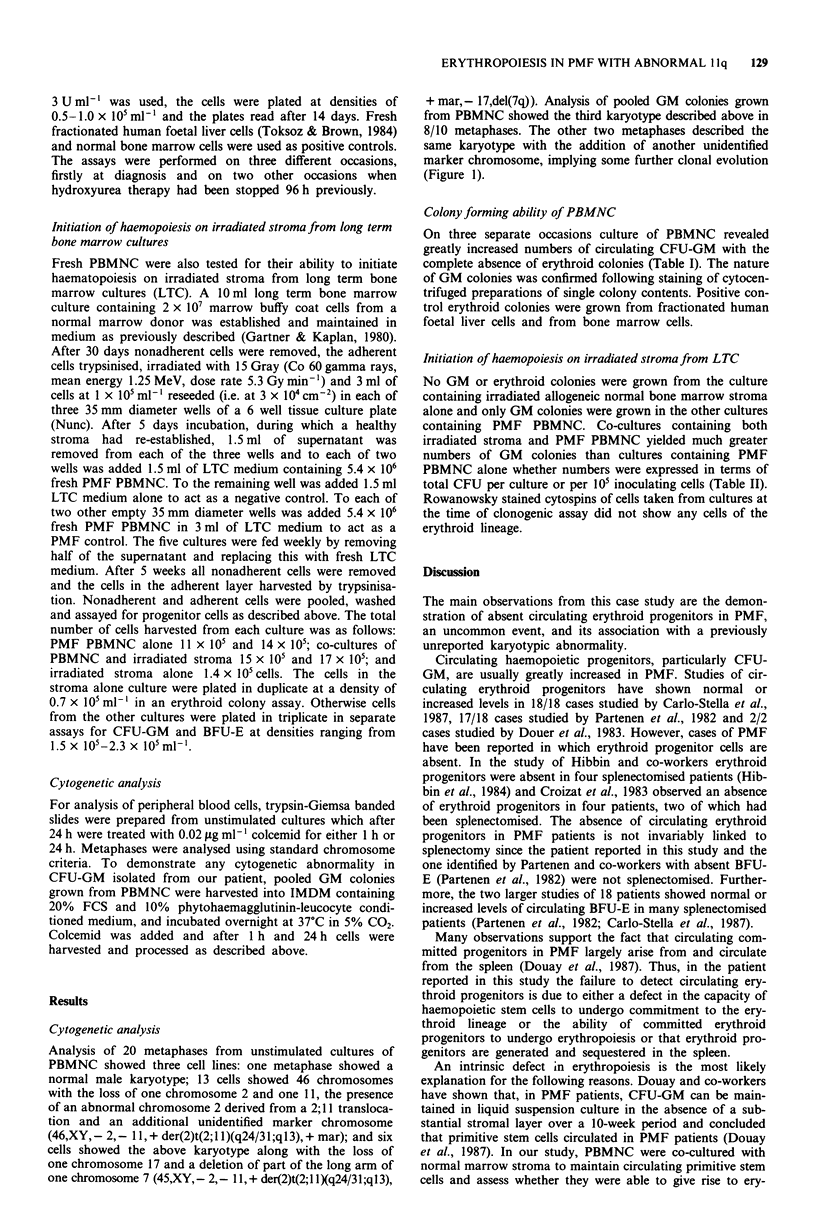

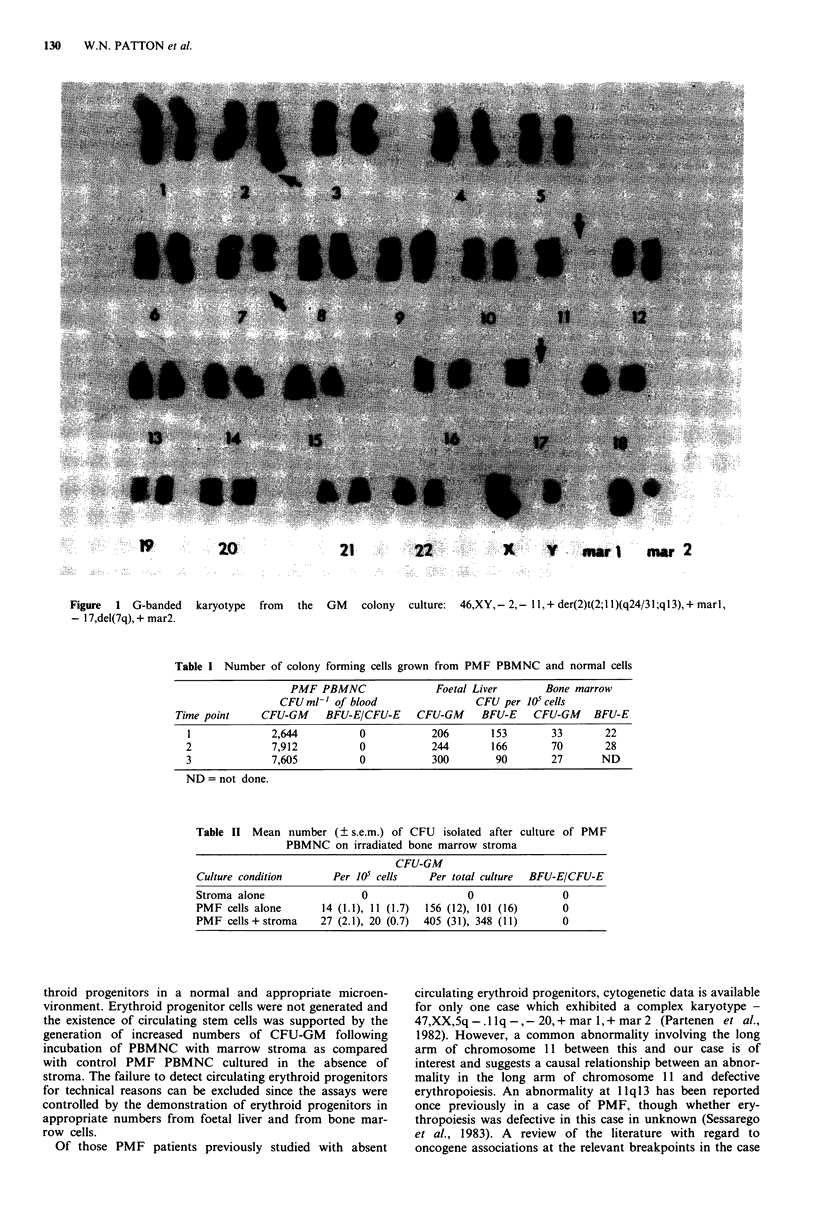

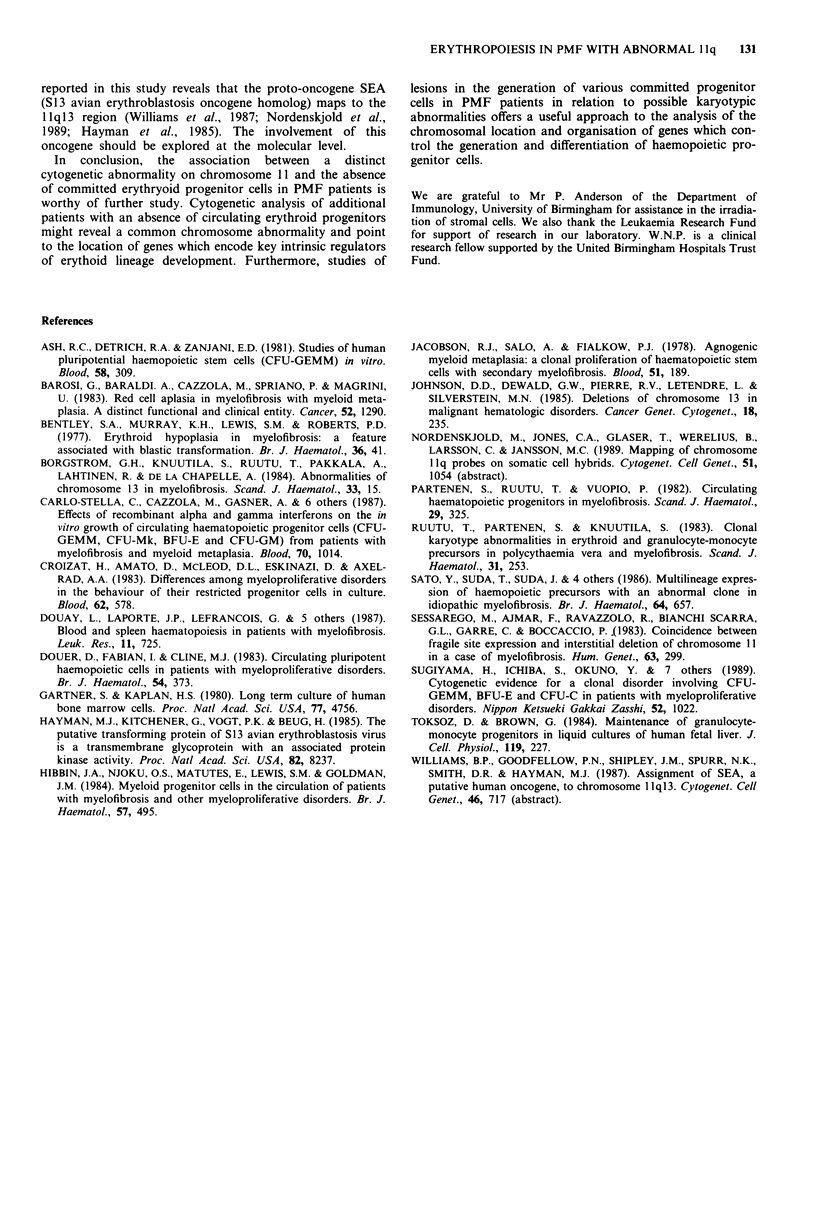


## References

[OCR_00366] Ash R. C., Detrick D. A., Zanjani E. D. (1981). Studies of human pluripotential hemopoietic stem cells (CFU-GEMM) in vitro.. Blood.

[OCR_00371] Barosi G., Baraldi A., Cazzola M., Spriano P., Magrini U. (1983). Red cell aplasia in myelofibrosis with myeloid metaplasia. A distinct functional and clinical entity.. Cancer.

[OCR_00375] Bentley S. A., Murray K. H., Lewis S. M., Roberts P. D. (1977). Erythroid hypoplasia in myelofibrosis: a feature associated with blastic transformation.. Br J Haematol.

[OCR_00379] Borgström G. H., Knuutila S., Ruutu T., Pakkala A., Lahtinen R., de la Chapelle A. (1984). Abnormalities of chromosome 13 in myelofibrosis.. Scand J Haematol.

[OCR_00384] Carlo-Stella C., Cazzola M., Gasner A., Barosi G., Dezza L., Meloni F., Pedrazzoli P., Hoelzer D., Ascari E. (1987). Effects of recombinant alpha and gamma interferons on the in vitro growth of circulating hematopoietic progenitor cells (CFU-GEMM, CFU-Mk, BFU-E, and CFU-GM) from patients with myelofibrosis with myeloid metaplasia.. Blood.

[OCR_00393] Croizat H., Amato D., McLeod D. L., Eskinazi D., Axelrad A. A. (1983). Differences among myeloproliferative disorders in the behavior of their restricted progenitor cells in culture.. Blood.

[OCR_00397] Douay L., Laporte J. P., Lefrancois G., Najman A., Dupuy-Montbrun M. C., Lopez M., Giarratana M. C., Gorin N. C. (1987). Blood and spleen haematopoiesis in patients with myelofibrosis.. Leuk Res.

[OCR_00402] Douer D., Fabian I., Cline M. J. (1983). Circulating pluripotent haemopoietic cells in patients with myeloproliferative disorders.. Br J Haematol.

[OCR_00407] Gartner S., Kaplan H. S. (1980). Long-term culture of human bone marrow cells.. Proc Natl Acad Sci U S A.

[OCR_00411] Hayman M. J., Kitchener G., Vogt P. K., Beug H. (1985). The putative transforming protein of S13 avian erythroblastosis virus is a transmembrane glycoprotein with an associated protein kinase activity.. Proc Natl Acad Sci U S A.

[OCR_00417] Hibbin J. A., Njoku O. S., Matutes E., Lewis S. M., Goldman J. M. (1984). Myeloid progenitor cells in the circulation of patients with myelofibrosis and other myeloproliferative disorders.. Br J Haematol.

[OCR_00423] Jacobson R. J., Salo A., Fialkow P. J. (1978). Agnogenic myeloid metaplasia: a clonal proliferation of hematopoietic stem cells with secondary myelofibrosis.. Blood.

[OCR_00428] Johnson D. D., Dewald G. W., Pierre R. V., Letendre L., Silverstein M. N. (1985). Deletions of chromosome 13 in malignant hematologic disorders.. Cancer Genet Cytogenet.

[OCR_00440] Partanen S., Ruutu T., Vuopio P. (1982). Circulating haematopoietic progenitors in myelofibrosis.. Scand J Haematol.

[OCR_00445] Ruutu T., Partanen S., Knuutila S. (1983). Clonal karyotype abnormalities in erythroid and granulocyte-monocyte precursors in polycythaemia vera and myelofibrosis.. Scand J Haematol.

[OCR_00453] Sato Y., Suda T., Suda J., Ohsaka A., Kubota K., Saito M., Miura Y. (1986). Multilineage expression of haemopoietic precursors with an abnormal clone in idiopathic myelofibrosis.. Br J Haematol.

[OCR_00456] Sessarego M., Ajmar F., Ravazzolo R., Bianchi Scarrà G. L., Garrè C., Boccaccio P. (1983). Coincidence between fragile site expression and interstitial deletion of chromosome 11 in a case of myelofibrosis.. Hum Genet.

[OCR_00462] Sugiyama H., Ichiba S., Okuno Y., Takahashi T., Imura H., Nakamura K., Iho S., Hoshino T., Okada T., Furukawa H. (1989). Cytogenetic evidence for a clonal disorder involving CFU-GEMM, BFU-E and CFU-C in patients with myeloproliferative disorders.. Nihon Ketsueki Gakkai Zasshi.

[OCR_00468] Toksoz D., Brown G. (1984). Maintenance of granulocyte-monocyte progenitor cells in liquid cultures of human foetal liver.. J Cell Physiol.

